# Nest-site selection in a fish species with paternal care

**DOI:** 10.1007/s10750-020-04470-0

**Published:** 2020-12-04

**Authors:** Theo C. M. Bakker, Beat Mundwiler

**Affiliations:** 1 Abteilung Verhaltensökologie, Zoologisches Institut, University of Bern, Wohlenstrasse 50a, 3032 Hinterkappelen, Switzerland; 2 Institute for Evolutionary Biology and Ecology, University of Bonn, An der Immenburg 1, 53121 Bonn, Germany

**Keywords:** *Gasterosteus aculeatus*, Three-spined stickleback, Dissolved oxygen concentration, Water temperature, Microhabitat, Male nest-site preference

## Abstract

Fish that perform paternal care may increase their fitness by choosing nest sites that enhance survival and development of embryos. We studied nest-site choice with respect to dissolved oxygen concentration and water temperature in males of the three-spined stickleback (*Gasterosteus aculeatus*), a small fish species with exclusive male parental care that usually breeds in the littoral zone of freshwaters of the Northern hemisphere. Fathers oxygenate the embryos by fanning movements of their pectoral fins. We expected choice for conditions at potential nest sites that would benefit offspring development, i.e., higher temperature and higher dissolved oxygen concentration. In the laboratory, we offered males a choice between two potential nest sites that differed in dissolved oxygen concentration or water temperature. Males preferred to build a nest at sites with a higher dissolved oxygen level or higher temperature and thus chose sites that would promote embryo development.

**Electronic supplementary material:**

The online version of this article (10.1007/s10750-020-04470-0) contains supplementary material, which is available to authorized users.

## Introduction

Nest-site choice has been particularly well studied in birds (see Barber, 2013) and is related to food availability and predator avoidance (e.g., Eichholz & Elmberg, 2014). It is assumed to be adaptive but there may be incongruence between nest-site preferences and nest success due to various anthropogenic, methodological, or ecological-evolutionary reasons such as limited access to nest sites due to competition, spatial variation in selection pressures, trade-offs with others selective pressures like microclimate, access to food, and extrapair copulations (reviewed in Chalfoun & Schmidt, 2012). Nevertheless, at the individual level, microhabitat nest-site choice may be crucial for the survival and development of progeny, especially in fishes as there is often considerable spatial variation in temperature and dissolved oxygen concentration (DOC) at potential nest sites. In addition to the water temperature and DOC, there are many abiotic factors that may direct nest-site choice such as risk of desiccation, wave exposure, and light intensity (e.g., Whoriskey & FitzGerald, 1994). Factors may act in concert or may be traded off among each other.

Among fishes that produce demersal eggs, male parental care is prevailing when there is external fertilization (Ridley, 1978; Gross & Sargent, 1985). Typically, in these species, the male builds a nest or occupies a burrow that serves as nest site. Females are attracted to spawn in the nest. Paternal care consists of nest defense against egg predators, which are often conspecifics, and direct care for the eggs by fanning the eggs with oxygenated water and removing diseased eggs (Ridley, 1978). Care may be extended for some time after hatching.

Fanning, i.e., the ventilation of eggs by driving a current of water over them, is commonly performed by movements of the pectoral fins (Jones & Reynolds, 1999a; van Lieshout et al., 2013). It is essential for the supply of oxygen for the developing embryo. The amount of fanning is adjusted to the oxygen requirements of the eggs: it increases with more advanced developmental stage, larger number of eggs in the nest, lower dissolved oxygen level, and higher temperature (van Iersel, 1953; Morris, 1954; Sevenster, 1961; Barlow, 1964; Reebs et al., 1984; Torricelli et al., 1985; Coleman & Fischer, 1991; Jones & Reynolds, 1999a). Both direct and indirect care for eggs is energetically costly (Sargent & Gebler, 1980; Sargent, 1985; Chellappa et al., 1989; Lindström & Hellström, 1993; Smith & Wootton, 1995a; von Hippel, 2000). For example, for species of fish that fan their eggs to oxygenate them there is substantial energy expenditure on parental care (Smith & Wootton, 1995a, b, 1999), apart from loss in condition due to limited foraging opportunities during the period of care. This may have consequences for future reproductive success (Sabat, 1994). In the common goby, *Pomatoschistus microps* (Krøyer, 1838), for example, males in the low oxygen treatment (about 35% DOC) lost more body mass than control fish during the first spawning. They were more likely to stop paternal care during the second spawning (Jones & Reynolds, 1999a). And in the sand goby, *Pomatoschistus minutus* (Pallas, 1770), males in the low oxygen treatment (about 40% DOC) lost more body fat, indicating a greater fanning effort (Lissåker et al., 2003). Thus, low oxygen concentration at the nest not only directly impairs embryo development and survival but indirectly also has an impact on the caring male that has to work harder (fan more) to supply the developing embryos with sufficient oxygen. In this way, low oxygen level may to some extent be compensated by male behavior.

There are several examples of microhabitat nest-site choice with respect to dissolved oxygen concentration or water temperature in fishes with paternal care. For example, bluegill sunfish, *Lepomis macrochirus* Rafinesque, 1819, inhabiting ponds with spatially variable dissolved oxygen levels chose well-oxygenated sites for nest building (Gosch et al., 2013). In another population of *L. macrochirus*, dissolved oxygen level was not significantly associated with nest sites but water surface temperature was (Stahr et al., 2013).

In addition to natural selection on nest-site choice due to food availability, predation on males and offspring, and the effects of oxygen and temperature on offspring development and survival, nest-site choice is under sexual selection pressure due to male–male competition for good territories, and female choice for nest sites.

We studied nest-site preference in three-spined sticklebacks (*Gasterosteus aculeatus* Linnaeus, 1758), a fish with exclusive male parental care (Wootton, 1976). Males are territorial and build a tunnel-shaped nest of plant material in the littoral zone of fresh and brackish waters of the Northern hemisphere (Wootton, 1976). There is male–male competition for territories (van den Assem, 1967). Males may collect eggs of up to 30 or even more females in the nest (Bakker et al., 2016) and care for the eggs by fanning that can make up to two-thirds of a male’s time-budget (van Iersel, 1953). Factors that stickleback males consider for nestbuilding are substrate (Rowland, 1994; Feller et al., 2016), water current velocity (Rushbrook et al., 2010), water depth (Kynard, 1979; Kraak et al., 2000; Vines & Schluter, 2006; Bolnick et al., 2015), and concealment by, e.g., vegetation (Jenni, 1972; Kynard, 1978, 1979; Mori, 1993; Candolin & Voigt, 1998; Kraak et al., 1999, 2000). The aim of the present study was to assess nest-site choice with respect to natural variation in temperature and dissolved oxygen concentration. Our expectation was that males would have a preference for nest sites that would benefit offspring development that is for sites with higher temperature or higher dissolved oxygen concentration.

## Materials and methods

**Fig. 1:**
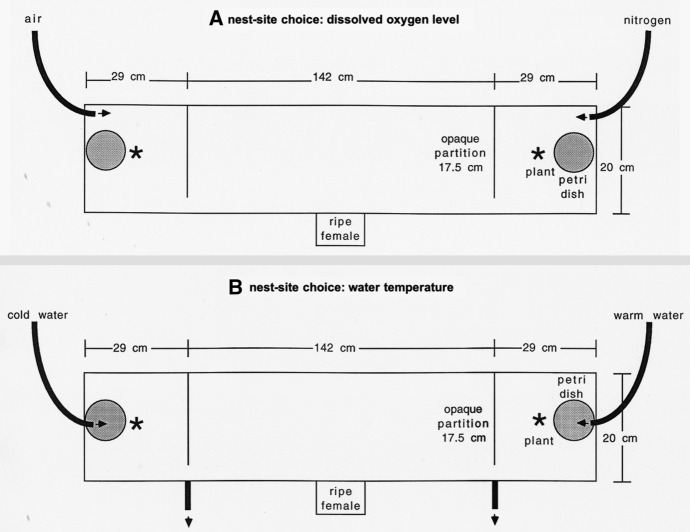
Bird’s-eye view of the experimental tank to test nest-site preferences with respect to **A** dissolved oxygen concentration and **B** water temperature. Two available nest sites (petri dish filled with sand and available filamentous algae) were offered at opposite ends of a 2 m-long tank that differed in DOC or water temperature. Differences were created by inflow of air or nitrogen gas and of cold or warm water, respectively. Males were stimulated by the presentation of a gravid female in front of the tank

Male nest-site choice with respect to dissolved oxygen concentration and water temperature was tested in the laboratory in choice experiments with two available nest sites that either differed in dissolved oxygen level or in water temperature (Fig. 1A, B). A tank of 200 × 20 cm, water level 19 cm, was divided in three compartments by gray opaque partitions placed at 29 cm from the small sides of the tank. The partitions left an opening of 2.5 cm at the front wall, so that fish could freely move between compartments. The left and right compartments were potential nest sites because they were provided with nesting substrate (a petri dish with sand, placed against the side wall halfway between the front and back wall), a longleafed plant (*Vallisneria* sp.; planted in a cup filled with coarse gravel and placed next to the petri dish at the side of the partition), and some tufts of green, filamentous algae for nest building. The middle compartment was illuminated by a 33 W fluorescent tube mounted 4 cm above the water surface. In order to avoid disturbance during nest-site choice and nest building, the tank was placed behind an opaque, dark green curtain. The tank was situated in an airconditioned room with simulated summer conditions (15°C, 16 h of illumination).

Test males had been sampled as juveniles from the Wohlensee (Lake Wohlen, the dammed-up river Aare near Bern, Switzerland, 46° 57′ N, 7° 28′ E) population during autumn 1992. They were stocked under simulated winter conditions (8-10°C, 8 h of illumination) in a group of several hundreds of fish of mixed sexes in a large tank with continuous inflow of fresh tapwater. In the summer of 1993, they were transferred to simulated summer conditions (15°C, 16 h of illumination). Under these conditions, males started to develop some breeding coloration but fish density prevented sexual activity. Experiments were performed between late summer of 1993 and early winter of 1994. The normal breeding season is from April to July (e.g., Kraak et al., 1999) but its occurrence can be manipulated by manipulation of the daylength (e.g., Bakker, 1986) without known behavioral effects. Males with developing breeding coloration were selected from the stock tank and put into the experimental tank. Males were given the opportunity to build a nest. When no nestbuilding activities were shown in 1–3 days, they were removed and replaced by another male. 11 Males built a nest, whereas 5 (31.3%) did not. At the end of the experiment, they were marked by clipping the tip of one of the spines and returned to the stock tank. Males were used only once. The fish were daily fed with live *Tubifex* sp. worms and defrosted *Chironomus* sp. larvae. Males in the test tank were fed late in the afternoon in the middle of the tank.

Differences in DOC between the two nest sites were created by inflow of nitrogen gas. This has become common practice for lowering DOC in behavioral studies with fish as it has no known side-effects on behavior (Jones & Reynolds, 1999a; Lissåker et al., 2003; Lindström et al., 2006; Pike et al., 2007; Head et al., 2017). Nitrogen gas was added via an air-stone located in the back corner of the tank. The other nest-site compartment was aerated via an air-stone with atmospheric air (Fig. 1A). Both air-stones released about an equal number of bubbles. Oxygen-rich and -poor nest sites were alternated between successive test males. As soon as a stable oxygen gradient had been established (after about one hour), the test male was introduced in the middle of the test tank and left undisturbed until completion of a nest at one of the two nest sites. The nitrogen inflow was adjusted such that DOCs were about 8 mg/l (84%) and 5 mg/l (51%) at the rich and poor nest site, respectively, thus within variation in the field (see Supplementary material). DOC and temperature were measured at the potential nest sites, i.e. above the petri-dishes with sand. Males may have sampled DOC and temperature at places in the tank where the differences were more pronounced. Oxygen concentrations were daily checked, and when necessary the nitrogen inflow was adjusted so that a relatively stable difference between nest sites was maintained until the male completed nest building. In order to promote nestbuilding, the male was stimulated with a ripe Wohlensee female enclosed in a 1-l plexiglass cell that was placed at the middle before the front wall from 9 a.m. till 6 p.m. Care was taken that for necessary activities (feeding, oxygen and temperature measurements, exchange of female), the experimental tank was approached as often from the left as from the right side.

In another experiment, we measured nest-site choice with respect to water temperature using a similar set-up and males from the same stock that were treated similarly. The air-stones were replaced by an inlet of cold and warm water, respectively, above the petri-dishes in the nesting compartments. An overflow near the partitions kept the water level in the tank constant (Fig. 1B). At one side, cold water (tap water cooled with cooling elements; temperature ranged from 12.1 to 12.9°C) from an aerated storage tank was dropped in at a rate of 20 ml/min, at the other side warm water (heated tap water; temperature ranged from 38.0 to 42.0°C) from an aerated storage tank was dropped in at the same rate. As soon as a stable temperature gradient had been established (after about half an hour), the test male was introduced in the middle of the test tank and left undisturbed until completion of a nest at one of the two nest sites or was removed after 1-3 days when no nestbuilding activities were shown. The water flow was stopped late in the afternoon and re-established early the next morning. The set-up was able to create a stable difference of about 0.5°C in water temperature between the nest sites, thus well within variation in the field (see Supplementary material). The experimental test is conservative as spatial variation in temperature can be greater than 2°C in the field (TCMB et al., unpubl. data). 21 Males built a nest, whereas 30 (58.8%) did not.

The tests for water temperature were run before the tests for dissolved oxygen. All tests were done in two test tanks that run parallel. Due to logistic reasons, the sample size of the tests for dissolved oxygen was smaller than that of the tests for water temperature (11 and 21, respectively).

### Data analysis

The significance of nest-site preferences was tested with binomial tests. DOC and water temperature were normally distributed according to Kolmogorov–Smirnov tests with Lilliefors correction. The difference in DOC and water temperature between nest sites were tested with paired *t* tests. Temporal patterns of DOC and temperature as well as the correlation between DOC and temperature were tested with regression analysis. All analyses were performed using R version 3.6.2 (R Core Team, 2019). Reported *P* values are two-tailed throughout.

## Results

**Fig. 2:**
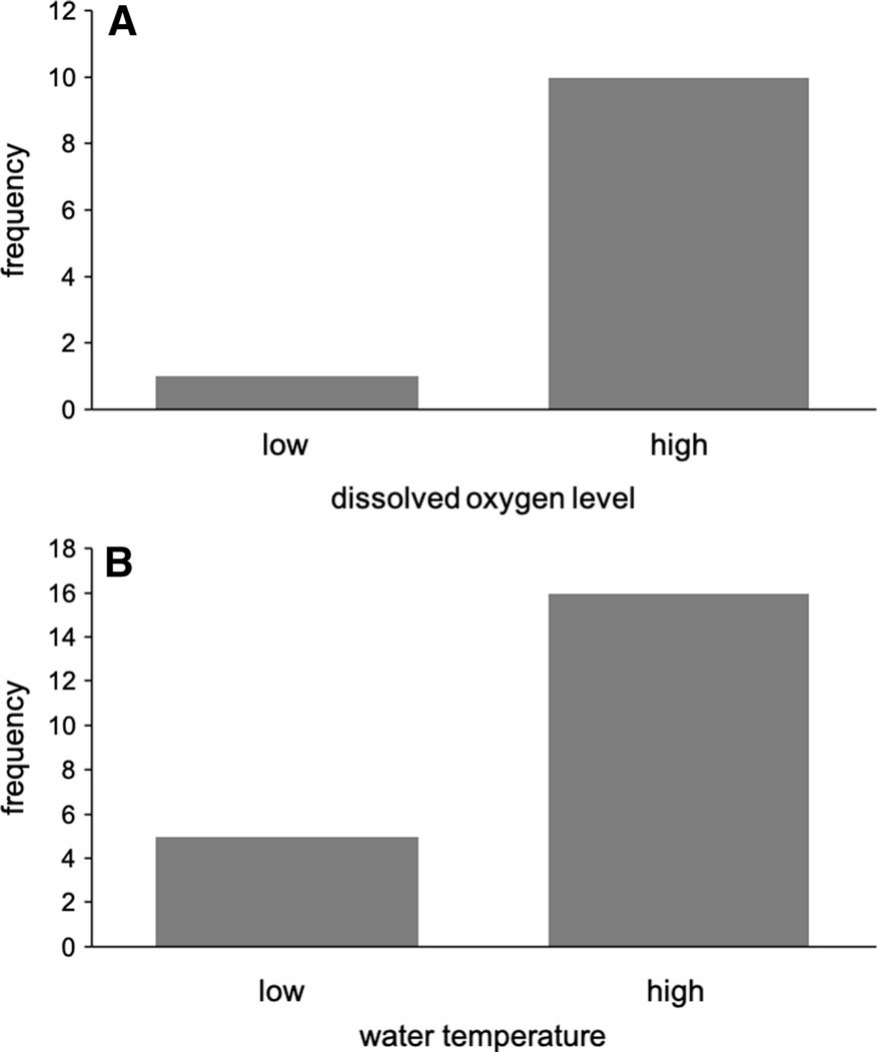
Frequency distributions of nest-site choice for sites with **A** high or low dissolved oxygen concentration (*N* = 11) and **B** high or low water temperature (*N* = 21)

Males clearly preferred to build their nest at the site with the higher dissolved oxygen concentration: 10 nests at the oxygen-rich site and 1 at the oxygen-poor site (binomial test, *N* = 11, *P* = 0.012; Fig. 2A). The median number of days used for nest building was 1 (range 1–4). The oxygen concentrations (mean ± SD) at the start of the experiment were 8.33 ± 1.18 mg/l (85.36 ± 11.05%) and 5.27 ± 1.81 mg/l (54.27 ± 18.04%) at the rich and poor sites, respectively (paired *t* test, df = 10, *t* = 13.36, *P* < 0.001). At the end of the first day of the males in the experimental tank, the levels were 7.83 ± 0.57 (80.55 ± 5.70%) and 4.15 ± 0.60 mg/l (43.00 ± 6.08%), respectively (paired *t* test, df = 10, *t* = 18.29, *P* < 0.001). The water temperature was not significantly different between the two nesting compartments (at start: oxygen-rich site 13.45 ± 0.35°C, oxygen-poor site 13.46 ± 0.35°C, paired *t* test, df = 10, *t* = 0.56, *P* > 0.58; at end of day: 13.50 ± 0.44°C and 13.50 ± 0.40°C, respectively, *t* = 0, *P* = 1).

Males also preferred the warmer nest site: 16 nests at the warmer site, 5 at the cooler (binomial test, *N* = 21, *P* = 0.026; Fig. 2B). Males that built at the cooler site were distributed evenly over the testing period. The median number of days used for nest building was 2 (range 1–3). The water temperature (mean ± SD) at the start of the experiment was 14.48 ± 0.37°C and 14.00 ± 0.30°C at the warm and cool sites, respectively (paired *t* test, df = 20, *t* = 8.78, *P* < 0.001). At the end of the first experimental day the temperature was 15.30 ± 0.63°C and 14.94 ± 0.48°C, respectively (paired *t* test, df = 20, *t* = 8.23, *P* < 0.001). The dissolved oxygen concentrations were not significantly different between the two nest sites (at start: warmer site 9.06 ± 0.78 mg/l (95.29 ± 7.59%), cooler site 9.20 ± 0.69 mg/l (95.14 ± 6.22%), paired *t* test, df = 20, *t* = 1.74, *P* > 0.097; at the end of first day: 9.20 ± 0.72 mg/l (97.81 ± 5.84%) and 9.07 ± 0.58 mg/l (95.86 ± 5.44%), respectively, paired *t* test, df = 20, *t* = 1.33, *P* > 0.19).

## Discussion

Stickleback males preferred to build their nests at sites with higher dissolved oxygen concentration and with higher temperature. Both abiotic factors were within the normal range during the breeding season of this fish population: at a fixed reference point in the Wohlensee, temperature ranged between 10.1 and 18.9°C and dissolved oxygen concentration between 5.0 and 10.4 mg/l (see Supplementary material). Higher temperature translates into faster embryo development (van Iersel 1953; Heuts, 1956; Wootton, 1976), which may be advantageous as the egg stage is very vulnerable to predation by con- and heterospecifics (Whoriskey & FitzGerald, 1994). One-degree higher temperature effects about half a day earlier hatching (van Iersel, 1953). The reported natural temporal variation in temperature was much higher than the temperature difference in the experiments but in nature the simultaneous choice in temperature among potential nest sites will be much smaller than the variation in temperature over the breeding season. So, the small difference is relevant to nest-site choice and is conservative as spatial variation in water temperature at nest sites in the field is greater than that in the experiments. In the experiments though, greater differences in temperature may be perceived as the difference of the inlet water temperature was much higher than that at the potential nest sites. Higher DOC may be beneficial as it also promotes embryo development and survival (Glippa et al., 2017) and may relieve paternal effort (e.g., van Iersel, 1953; Sevenster, 1961). DOC and temperature were not significantly correlated in the field (*r*^2^ = 0.016, *P* = 0.372; see Supplementary material, Fig. S1) due to variation in hydro-meteorological conditions and the intensity of biological processes (Rajwa-Kuligiewicz et al., 2015). Thus, males have to assess both abiotic factors in the field to optimize nest-site choice. In addition to spatial microhabitat variation in DOC and temperature (Kraak et al., 1999; TCMB and BM unpubl. data), there existed temporal variation in the abiotic factors (temperature: linear increase, *r*^2^ = 0.618, *P* < 0.001, Fig. S2A; DOC: non-linear change, *r*^2^ = 0.425, *P* < 0.001, Fig. S2B). This may have repercussions for nest-site selection over time as early in the breeding season temperature may be a limiting factor for embryo development but in the middle of the season rather DOC may become a limiting factor.

Adult sticklebacks and stickleback embryos are relative tolerant to low DOC (Moran et al., 2010; TCMB et al., unpubl. data). In sticklebacks, as in other fishes (e.g., brook trout *Salvelinus fontinalis* (Mitchill, 1815) and rainbow trout *Salmo gairdneri* (Richardson, 1836) (Garside, 1966), black bream *Acanthopagrus butcheri* (Munro, 1949) (Hassell et al., 2008)), lower DOC retards embryo development and decreases hatching rate (Moran et al., 2010; Glippa et al., 2017; TCMB et al., unpubl. data). The effects of water temperature on embryo development and survival seem stronger at least at low temperatures (e.g., Garside, 1966; TCMB et al., unpubl. data). Embryo mortality increases with higher temperature (Hopkins et al., 2011; Shama & Wegner, 2014; Shama et al., 2014; TCMB et al., unpubl. data). There thus is potential for studying the interplay of DOC and temperature in nest-site choice. Future studies may investigate these two factors in a factorial design. Due to climate change, fishes will be increasingly confronted with higher water temperature and lower DOC during summer (Missaghi et al., 2017), which may influence nest-site choice. In nature, biotic factors like male–male competition and predation risk may further direct nest-site choice.

Our results are in agreement with indications that DOC and temperature play a role in nest-site choice in sticklebacks and other nestbuilding fishes. In a Swiss population of three-spined sticklebacks in Roche (near Montreux), Kraak et al. (1999) showed that in May there was a correlation of temperature at the nest site with mating success whereas in June standardized DOC at the nest had a nonsignificant positive effect on mating success (multiple regression with significant effects of body size and presence of a plant at the nest). It is unclear whether these correlations are a consequence of male and/or female preferences for particular nest sites and/or female preferences for males that nest at particular sites and/or for their nests (males built more compact nests at high DOC: Head et al., 2017). There is need for further research to disentangle these factors. In the present study, we only measured male preferences. Preliminary tests pointed to an effect of DOC on female preferences for nest sites in sticklebacks (TCMB et al., unpubl. data).

In several other fish species, females prefer nest sites with high DOC (Jones & Reynolds, 1999b; Reynolds & Jones, 1999; Smith et al., 2001; Payne et al., 2002). In the beaugregory damselfish *Stegastes leucostictus* (Müller & Troschel, 1848), a marine fish with male parental care (guarding, not fanning; Breder & Rosen, 1966), artificial spawning sites with low oxygen levels were avoided by spawning females (Payne et al., 2002). Females of the common goby, *P. microps*, a coastal marine fish with male parental care (guarding, fanning), preferred to spawn in nests with small entrances under saturated oxygen conditions but under low oxygen conditions the preference disappeared (Jones & Reynolds, 1999b). Nests with small entrances were detected less often by predatory shore crabs and small entrances may hamper egg ventilation by males. Under low DOC, males made larger nest entrances (Jones & Reynolds, 1999b). Several studies have shown that females of various fishes prefer to spawn in nests that already have egg clutches (see summary Table in Reynolds & Jones, 1999). However, in *P. microps*, this preference was reversed under low oxygen conditions (Reynolds & Jones, 1999): under low DOC, females preferred to spawn with males that had empty nests. This may have been due to changed male behavior (increased fanning activity, decreased time close to female; Reynolds & Jones, 1999) or female preference for sites with higher DOC. Females preference for nest sites with high DOC may interfere with preference for paternal or courtship fanning behavior (as in fifteen-spined sticklebacks, *Spinachia spinachia* (Linnaeus, 1758) Östlund & Ahnesjö, 1998). The bitterling (*Rhodeus sericeus* (Pallas, 1776)), a fish that lays its eggs on the gills of living freshwater mussels, showed adaptive female preference for mussel species and number of present fish embryos on the mussel gills (Smith et al., 2000, 2001; for the rose bitterling *R. ocellatus* (Kner, 1866), see Spence & Smith, 2013). Female choices were based on the oxygen conditions inside the mussel, which females deduced from the change in oxygen concentration between inhalant and exhalant siphons of a mussel (Smith et al., 2001).

Studies on temperature preferences for nest sites in males and/or female sticklebacks or other nestbuilding fishes are usually limited to the assessment of the temperature range suitable for spawning. Preference studies focused on nest depth and depth correlates with temperature at the nest site. Vines & Schluter (2006) showed that preferences for nest depth were different between limnetic-like and benthic-like three-spined stickleback males from different Canadian populations. In another Canadian stickleback population, body size, trophic morphology, and diet correlated with the depth of the nest (Snowberg & Bolnick, 2012). Nest depth was a strong predictor of male reproductive success (presence of eggs or fry in the nest) in four Canadian populations of sticklebacks: shallower nests were more likely to contain offspring. The effect of nest depth was stronger than the effects of male traits like coloration and body size (Bolnick et al., 2015). Depth of the nest may have had an effect on male reproductive success in a Swiss population early in the breeding season but depth was correlated with various factors that also correlated with reproductive success (Kraak et al., 1999). Early in the season, mating success was positively correlated with temperature at the nest indicating female preference for higher temperature or male traits associated with higher temperature at the nest in this population in May (Kraak et al., 1999). In the same Swiss population in June, depth level at the nest correlated with the blue intensity of the eyes of the nest owner and with nesting success but the latter correlation was confounded by nests hidden in a macrophyte (Kraak et al., 1999). Depth (temperature) may also influence the association between attractive male traits and other microhabitat characteristics at the nest site. Kraak et al. (2000) found in an experimental study, in which they created nest sites with or without macrophytes at different depths, that at the shallow depth redder and bigger males preferred to nest in a macrophyte whereas for males nesting at the deeper level, this was not the case. At the swallow depth, predation risk by birds was probably higher (Kraak et al., 2000).

In conclusions, in the present study, male three-spined sticklebacks preferred nest sites with higher temperature and higher DOC. There are indications from the literature that females may have similar preferences for microhabitat characteristics but these may be confounded by associations between microhabitat characteristics and attractive male traits and/or associations between microhabitat characteristics. Future research should aim at disentangling this complex of factors, which may shift with time in the breeding season. It will be a challenge to quantify fitness effects of microhabitat on nest-site choice in sticklebacks (Richardson et al., 2014).

## Electronic supplementary material

Below is the link to the electronic supplementary material.

Supplementary material 1 (PDF 112 kb)

## Data Availability

The datasets generated during this study are available from the corresponding author on reasonable request.
