# Supplementary material for: Nest-site selection in a fish species with paternal care
Source: Hydrobiologia. 2020 Dec 4;848(3):641–50. doi: 10.1007/s10750-020-04470-0 (PMC7822789; doi:10.1007/s10750-020-04470-0)
Supplement: Supplementary file 1 — Supplementary material 1 (PDF 112 kb) [file 10750_2020_4470_MOESM1_ESM.pdf]

Supplementary material
Hydrobiologia

Nest-site selection in a fish species with paternal care

Theo C. M. Bakker and Beat Mundwiler

Corresponding author: Theo C. M. Bakker, ORCID ID https://orcid.org/0000-0002-2797-2681
Institute for Evolutionary Biology and Ecology, University of Bonn, An der Immenburg 1, 53121 Bonn, Germany; email tbakker@evolution.uni-bonn.de

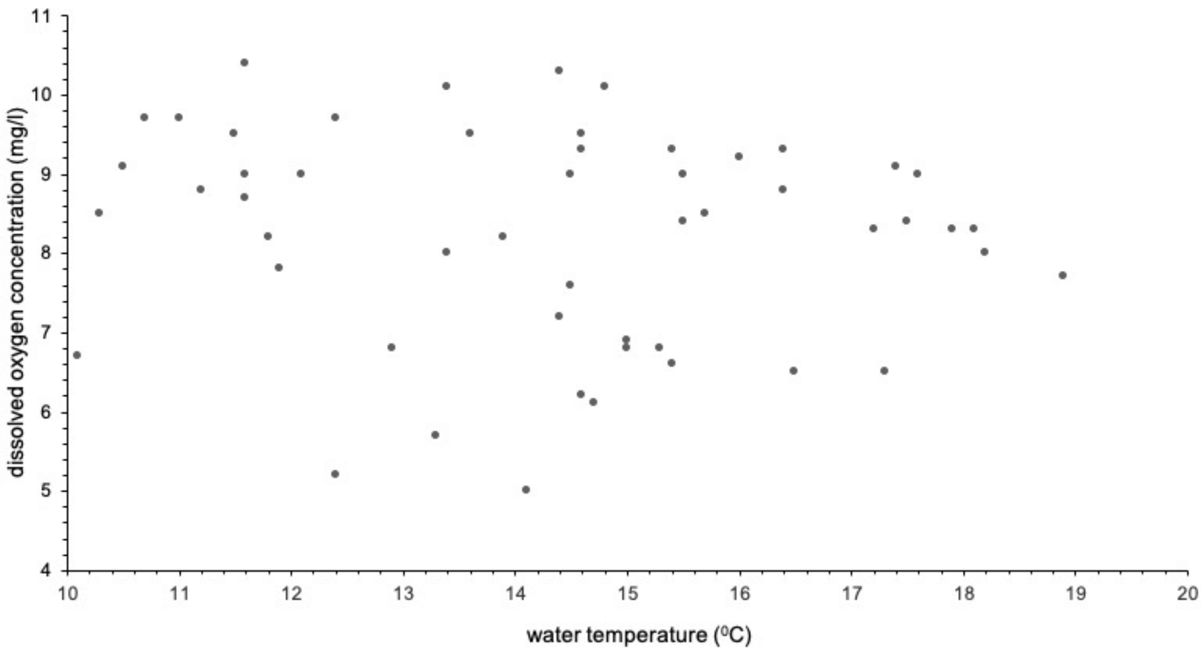

Fig. S1 Variation in water temperature (in ^{O}C) and dissolved oxygen concentration (in mg/l) in the Wohlensee near Bern during the 1993 breeding season (April-July). There existed no significant relationship between the two variables (r^2=0.016, N=52, p=0.372). Temperature and DOC were almost daily measured at a fixed reference point (low current velocity, close to the bank, depth about 10 cm) near the boathouse at the Institute using a WTW Oximeter OXI96 with an EOT 186-electrode. When measurements were taken more than once per day (in about half the cases), daily average values were calculated. Values ranged between 10.1 and 18.9^{0}C and 5.0 and 10.4 mg/l for water temperature and DOC, respectively

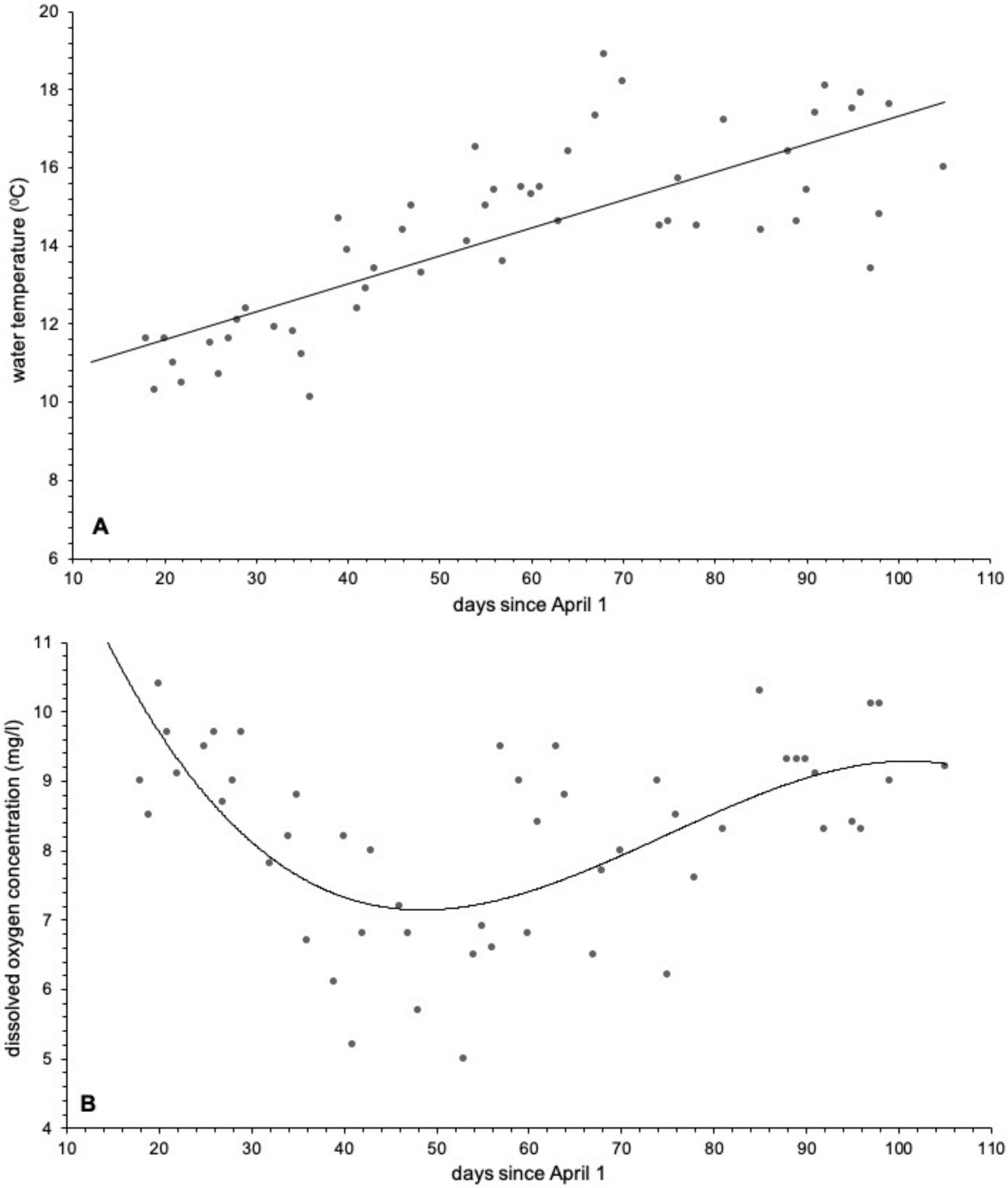

Fig. S2 Temporal (in days since April 1) change in **A)** water temperature (in ^{0}C) and **B)** dissolved oxygen concentration (in mg/l) at a fixed reference point in the Wohlensee near Bern during the 1993 breeding season (April-July). See the legend of Fig. S1 for further details. Water temperature increased significantly over the breeding season ($y = 0.0711x + 10.196$, $N = 52$, $r^2 = 0.618$, $p<0.001$) whereas DOC showed a non-linear relationship with time ($y = -0.00003x^3 + 0.0066x^2 - 0.433x + 15,957$, $N=52$, $r^2 = 0.425$, $p<0.001$)
